# Endurant Stent Graft for Treatment of Abdominal Aortic Aneurysm Inside and Outside of the Instructions for Use for the Proximal Neck: A 14-Year, Single-Center Experience

**DOI:** 10.3390/jcm13092589

**Published:** 2024-04-28

**Authors:** Giulio Accarino, Francesco De Vuono, Giancarlo Accarino, Giovanni Fornino, Aniello Enrico Puca, Rodolfo Fimiani, Valentina Parrella, Giovanni Savarese, Sergio Furgiuele, Carmine Vecchione, Gennaro Galasso, Umberto Marcello Bracale

**Affiliations:** 1 Vascular and Endovascular Surgery Unit, Ospedale San Giovanni di Dio e Ruggi D’Aragona, 84131 Salerno, Italy; giovanni.fornino@sangiovannieruggi.it (G.F.); dott.puca83@tim.it (A.E.P.); fimianirodolfo96@gmail.com (R.F.); va.parrella@gmail.com (V.P.); 2 Vascular Surgery Unit, Department of Public Health, University Federico II of Naples, 80138 Naples, Italy; dr.savarese@savasoft.net (G.S.); umbertomarcello.bracale@unina.it (U.M.B.); 3 Department of Medicine, Surgery and Dentistry, University of Salerno, 84084 Salerno, Italy; francescodevuono98@gmail.com (F.D.V.); gcaccarino@tin.it (G.A.); cvecchione@unisa.it (C.V.); ggalasso@unisa.it (G.G.); 4 Vascular Surgery Unit, Struttura Ospedaliera ad Alta Specialità Mediterranea, 80122 Naples, Italy

**Keywords:** EVAR, endoleak, long-term follow-up

## Abstract

**Aim**: To assess the medium and long-term performance of the Endurant stent graft in a cohort of consecutive patients treated with this device for an abdominal aortic aneurysm (AAA) both inside and outside of the instructions for use (IFU) and to find factors influencing the outcomes. **Methods**: Our observational, retrospective, single-center study included all patients who consecutively underwent endovascular aneurysm repair with the Endurant stent graft from February 2009 to January 2023. Patients with an AAA to treat according to current guidelines were included. Patients were divided into two groups: Group 1 inside of the IFUs and Group 2 outside of the IFUs for the proximal aortic neck. Patients were followed up after the procedure with computed angiography tomography, ultrasound examination, and interviews. Aneurysm-related mortality, procedure-related reinterventions, and type IA and III endoleaks were considered primary endpoints. Secondary endpoints included aneurysmal sac variations and graft thrombosis. **Results**: A total of 795 patients were included, 650 in Group 1 and 145 in Group 2; 732 were males, and the mean age was 74 ± 8. Anamnestic baseline did not differ between the two groups. Neck length, width, and angulation were different between the two groups (all *p* < 0.001). A total of 40 patients had a ruptured AAA, while 56 were symptomatic. At a mean follow-up of 43 ± 39 months, aneurysm-related mortality was less than 1%, and 82 endoleak (10.5%) were observed. Overall endoleak rate and type 1A endoleak, as well as procedure-related reintervention, were significantly more frequent in Group 2. Sac regression of at least 5 mm was observed in 65.9% of cases. AAAs larger than 60.5 mm carried a higher risk of endoleak (HR: 1.025; 95% CI: 1.013–1.37; *p* < 0.001) and proximal necks shorter than 13.5 mm carried a higher type 1A risk (HR: 0.890; 95% CI: 0.836–0.948; *p* < 0.001). Patients without chronic obstructive pulmonary disease and taking lipid-lowering drugs had an overall more consistent sac-shrinking rate. **Conclusions**: The Endurant stent graft proves safe and reliable. Out-of-IFU treatment has poorer medium and long-term outcomes. Some conditions influence medium and long-term reintervention risk and sac behavior. Patients with bigger aneurysms, proximal necks shorter than 13.5 mm, and chronic obstructive pulmonary disease should be more carefully evaluated during follow-up. Consistent follow-up is in keeping low aneurysm-related mortality. Personalized risk profiles and peri and postoperative management strategies are needed.

## 1. Introduction

Since the initial steps were taken in 1991 with the endovascular aneurysm repair (EVAR) technique [1], the treatment of abdominal aortic aneurysms (AAA) has evolved dramatically. More and more surgeons and patients prefer EVAR treatment over long and difficult open surgeries (OSR) since it is less invasive and allows for much faster recovery [2]. EVAR had a significant impact on the proportion of patients who were able to be treated for an AAA despite having concomitant medical issues that may have resulted in a high-risk OSR. These advantages come at the expense of EVAR’s lower durability when compared to OSR since there is a need for stricter follow-up and a higher reintervention rate [3,4]. Several stringent anatomical requirements must be satisfied for the selected device to be employed to deliver a safe and reliable EVAR treatment, which are commonly referred to as instructions for use (IFU). There are several endografts on the market now, each focusing on being more reliable in specific AAA anatomies, and most of them rely on proximal fixation either below or above the emergence of the renal arteries, as well as an exoskeleton internally tapered with a blood-tight fabric. Herein, we present our experience of 795 consecutive patients treated in our large-volume hub center in southern Italy using the Endurant (Medtronic Vascular, Santa Rosa, CA, USA) stent graft, both inside and outside the IFU, and report the follow-up data we collected, aiming at finding differences in outcomes between groups and other factors influencing medium and long-term outcomes.

## 2. Materials and Methods

We maintained a registry of consecutive patients treated for AAA with an Endurant stent graft between February 2009 and June 2023. All principles in the Declaration of Helsinki were followed, and the Italian laws on privacy (Art. 20–21, DL 196/2003) as published in the Official Journal, volume 190, 14 August 2004, which explicitly waives the need for ethical approval for the use of anonymous data, was respected. Our patients were retrospectively collected before October 2020, and since then, they have been prospectively collected. All patients gave written consent for the anonymous collection of clinical data on the standard consent form provided by our institution. Inclusion criteria were the presence of an AAA of surgical interest according to the Italian guidelines [5] (diameter of at least 50 mm or growth of more than 10 mm/year or symptomatic) and the use of an Endurant (I or II/IIs) endograft or an Endurant aortic cuff/tube.

Before the procedure, a Computed Tomography Angiography (CTA) was performed on our patients to assess the aorto-iliac anatomy and plan the procedure. Procedure planning was carried out with version 10.3 3mensio and measured by the same operator (Pie Medical Imaging, Maastricht, The Netherlands).

Patients’ demographics, comorbidities, and anatomic characteristics were identified and collected (Table 1). Patients were divided into two groups according to the anatomical suitability of their proximal aortic neck for the Endurant stent graft as per device (IFU) (at least 10 mm long, 19–30 mm wide, less than 60°-angled neck) or a neck length of at least 4 mm if used in conjunction with endoanchors. Their outcomes were compared, and any differences related to our endpoints were investigated. Patients treated inside the IFU are also referred to as “Group 1”, while patients treated outside of the IFU are also referred to as “Group 2”.

Patients with chimneys and monoiliac implants were included. All procedures were performed in the same dedicated operating theater equipped with a GE OEC 9900 Elite C-arm (GE Healthcare, Little Chalfont, UK) and by gaining surgical exposure of both common femoral arteries.

All procedures were performed using an Endurant I, II, or IIs stent graft. Device description and implantation technique are well-described elsewhere [6].

The proximal landing zone was planned to be as close as possible to the lower renal artery, polar renal artery sparing as well as the use of e embolizing spirals was discussed for each case and planned if there were at least 2 patent lumbar arteries with a diameter > 2 mm and a patent inferior mesenteric artery larger than 3 mm.

Procedural success was defined as the correct positioning of the device with conserved patency of the lower renal artery and the absence of high-flow endoleak (types I and III) at procedure completion. Operating theater occupation time was calculated considering all the time the patient stayed inside the operating room.

A follow-up CTA was not carried out on patients presenting an estimated glomerular filtration rate < 45 mL/min other than one month after the procedure due to the risk of acute renal failure. These patients were subsequently evaluated with duplex ultrasonography to identify aneurysmal sac variations and endoleak. Sac variation data were derived from CTAs and measured by the same operator to avoid interoperator differences.

All CTAs were evaluated by the same physician who did preoperative planning during patient follow-up.

Aneurysm-related mortality, procedure-related reinterventions, and high-flow (type 1 and 3) endoleaks were considered to be primary endpoints. Secondary endpoints included aneurysmal sac variations and graft thrombosis.

### Statistical Analysis

The normal distribution of continuous parameters was tested with the Kolmogorov–Smirnov and the Shapiro–Wilk tests. Normally distributed variables were expressed as mean ± standard deviation and compared using the student *t*-test; variables with a skewed distribution were reported as median and interquartile range and were compared with the Mann–Whitney U test. When appropriate, categorical variables were reported as numbers and percentages and compared using the χ2 test or Fisher exact test.

Receiver operating characteristic (ROC) curve analysis was performed to identify the best cut-off value of the maximum AAA diameter for the risk of any endoleak.

The association between the maximum AAA diameter and the risk of any endoleak was calculated using unadjusted and adjusted Cox proportional hazard regression models and presented as HR with their 95% confidence intervals (95%CI).

Survival free from the study outcomes was estimated using the Kaplan–Meier method. The Log-rank test was used to assess differences between the in and out of the IFU treatment groups.

For all tests, a *p*-value < 0.05 was considered statistically significant. Statistical analysis was performed using SPSS software version 29.0 (SPSS Inc., Chicago, IL, USA).

## 3. Results

A total of 795 patients treated with the Endurant stent graft were collected between February 2009 and January 2023 among the 1256 EVAR procedures performed in that period. A total of 650 were in the “in IFU” group, while 145 were in the “out of the IFU” group. All patients had an atherosclerotic aneurysm of surgical interest.

### 3.1. Baseline

Patients’ mean age was 74 ± 8 years, and 92% of them were male. The most prevalent comorbidities were arterial hypertension and chronic obstructive pulmonary disease, reported in 80 and 59.8% of patients, respectively. Baseline demographics and comorbidities are reported in Table 1. No statistically significant difference was observed regarding comorbidities in the two groups. Overall, the aneurysm size was 61 mm, bigger in Group 2 (*p* < 0.001); the aortic bifurcation diameter was also different among the two groups, showing a larger bifurcation in patients treated out of the IFU (*p* = 0.025). Small aneurysms (<15 mm) were 15%. Additional baseline anatomical characteristics are reported in Table 2. Proximal aortic neck features have been carefully evaluated and show a statistically significant difference in neck length, width, and angulation (all *p* < 0.001). All values regarding the proximal aortic neck, including calcium, thrombus, and overall neck morphology, have been evaluated and are reported in Table 3.

### 3.2. Periprocedural Data

Patients were hospitalized a mean of two days before surgery for preoperative diagnostic assessments, including routine blood tests and an echocardiographic exam. All procedures were performed in the same dedicated operating theater using a GE 9900 Elite (GE Healthcare, Ltd., Madison, WI, USA) C-arm and a powered injector. Patients who were operated on in elective settings were subjected to spinal anesthesia, while the patients who were operated on in emergency settings were treated under general anesthesia. A total of 40 patients were treated for a ruptured AAA, and 56 were treated for a symptomatic non-ruptured AAA. We gained bilateral surgical access to both common femoral arteries for all patients. Technical success was achieved in 99.25% of patients with only two intraoperative type 1A endoleaks, one from Group 1 and one from Group 2, and 4 deaths (3 in Group 1 and 1 in Group 2) in the first 24 h. The intraoperative type 1A in Group 1 was treated with an endoanchor implant during the same procedure. Procedural complications were reported in 6.3% of cases; in 35 patients, a patch angioplasty of one of the femoral arteries was required, while in 15 patients, there were complications related to the anesthesia or a small external iliac artery that required an unscheduled balloon angioplasty or stenting. A bell-bottom extension was used in 30% of patients. Sac embolization was required in a minority of patients (184). There were no statistically significant differences in periprocedural data between the two groups except for the length of stay, which was slightly higher in Group 2 (*p* = 0.01), and access-related complications, which were higher in Group 2 (*p* = 0.041). All periprocedural data are reported in Table 4.

### 3.3. Outcomes

**Figure 1:**
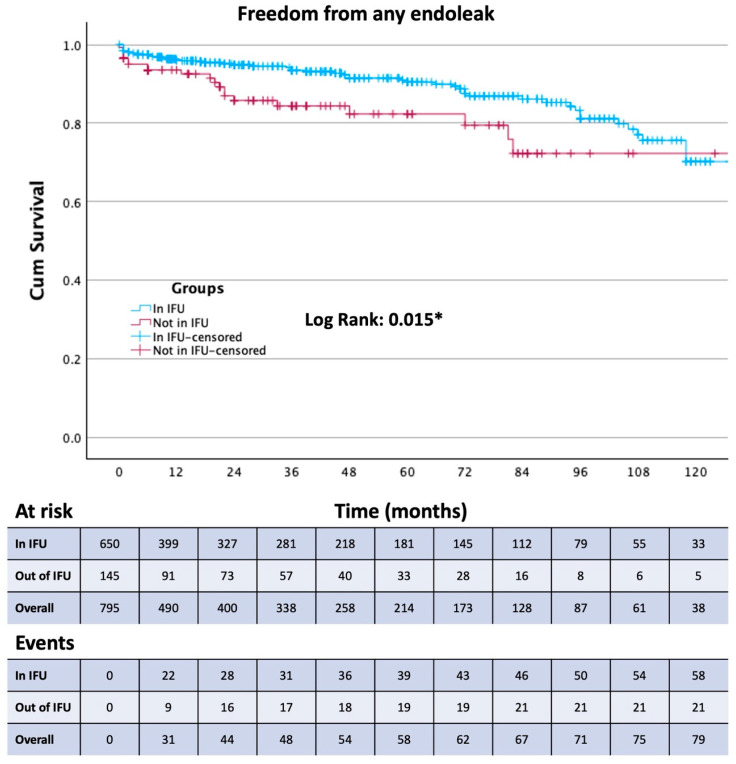
Kaplan–Meier curve showing Freedom from any endoleak in the two study groups. * Statistically significant (*p* < 0.05).

**Figure 2:**
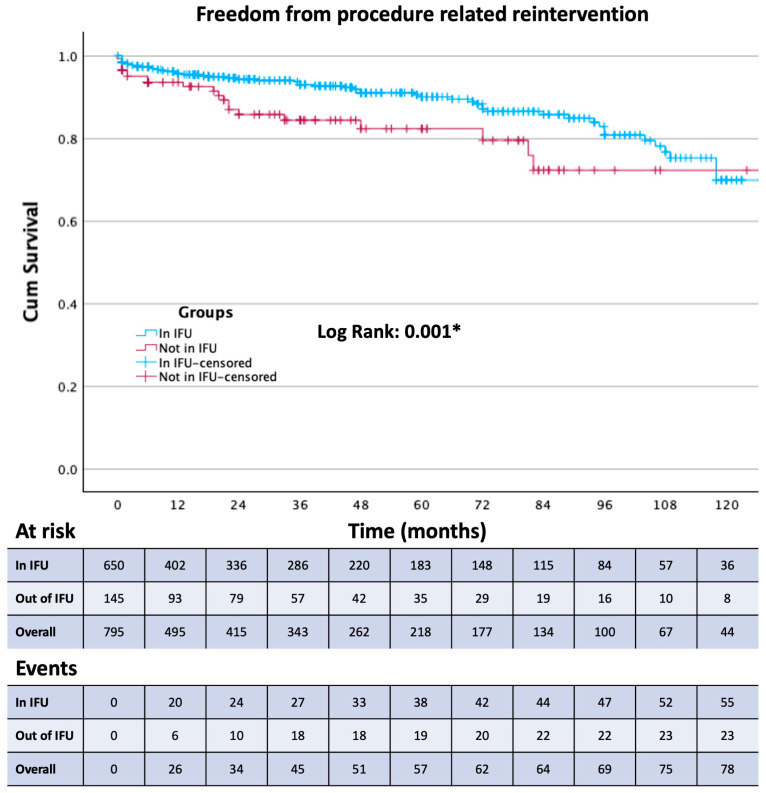
Kaplan–Meier curve showing Freedom from procedure-related reintervention in the two study groups. * Statistically significant (*p* < 0.05).

**Figure 3:**
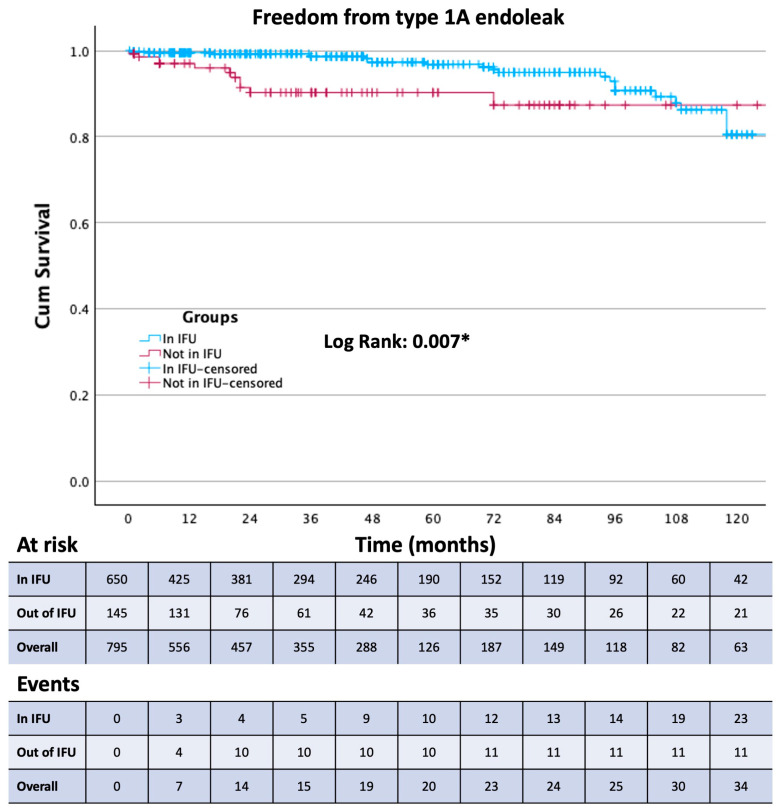
Kaplan–Meier curve showing Freedom from type 1A endoleak in the two study groups. * Statistically significant (*p* < 0.05).

**Figure 4:**
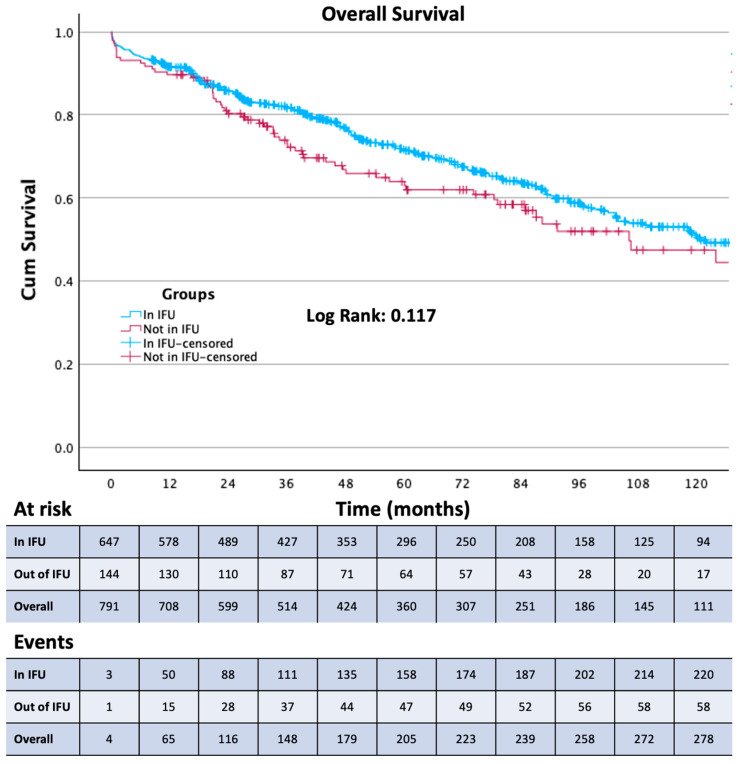
Kaplan–Meier curve showing overall survival in the two study groups.

Thirty-day mortality was observed in 29 patients, 20 from Group 1 and 9 from Group 2. Among these patients, 23 were treated in an emergency setting. Patients were followed up for a mean of 43 ± 39 months. The composite primary endpoint (aneurysm-related mortality, procedure-related reinterventions, and type IA and III endoleaks) was present in 122 patients. All outcomes are reported in Table 5. Aneurism-related mortality was reported in 9 patients at a mean of 61 ± 43 months. Of these 9 patients, 8 were lost at follow-up, and 5 presented to the emergency department with a ruptured AAA and died during the subsequent procedure or in the following days. The occurrence of any endoleak (Figure 1), the reintervention rate (Figure 2), and the type 1A endoleak (Figure 3) rate are all higher in Group 2 (*p* < 0.05). Two chimney procedures were performed on short necks. While they were both inside of the IFUs, one suffered from a gutter-related type 1A endoleak. Of the 17 patients who experienced graft thrombosis, 16 were subjected to a reintervention, while only 14 of the 30 type 2 endoleaks required reintervention. As only 18% of patients received sac embolization, the type 2 endoleak rate, in our experience, is fairly low. Sac embolization was performed with at least 3 controlled-release coils. Time-dependent outcome comparisons between the study groups are reported in Figure 1, Figure 2, Figure 3 and Figure 4. Interestingly, the rate of type 1B endoleaks in Group 2, selected to be outside of the IFU for the proximal aortic neck, is double that of Group 1, although not reaching statistical significance. Only one type 3 endoleak was reported in a patient lost at follow-up who presented with a ruptured AAA and died during emergency treatment.

**Figure 5:**
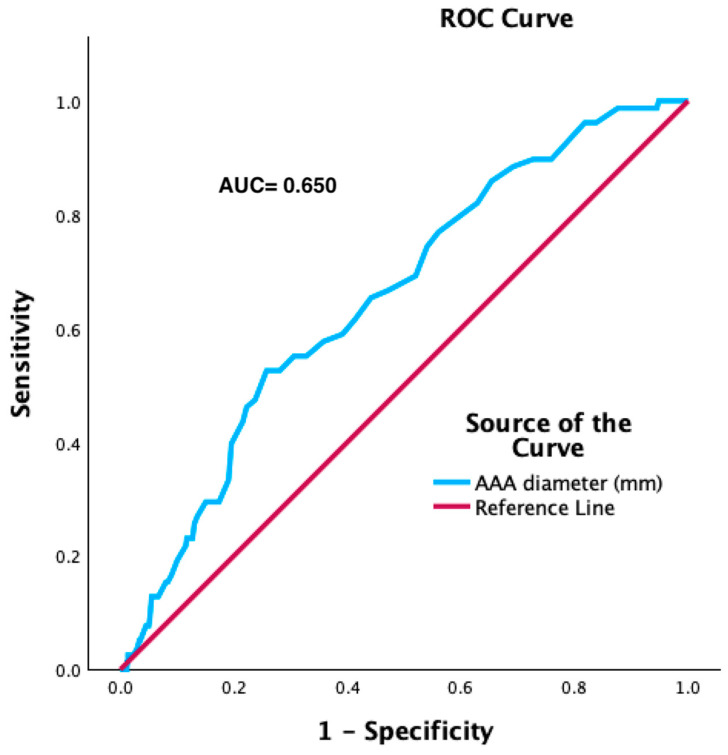
The ROC curve analysis with maximum AAA diameter is a predictive factor for any endoleak.

ROC curve analysis identified an aneurysm of a maximum diameter of 60.5 mm as the best cut-off value for predicting the risk of any endoleak during follow-up (AUC: 0.65; sensitivity: 61.5%; specificity: 41%) (Figure 5). At unadjusted Cox proportional hazard regression analysis, an AAA >60.5 mm was associated with a significantly higher risk of any endoleak (HR: 1.025; 95% CI: 1.013–1.37; *p* < 0.001).

**Figure 6:**
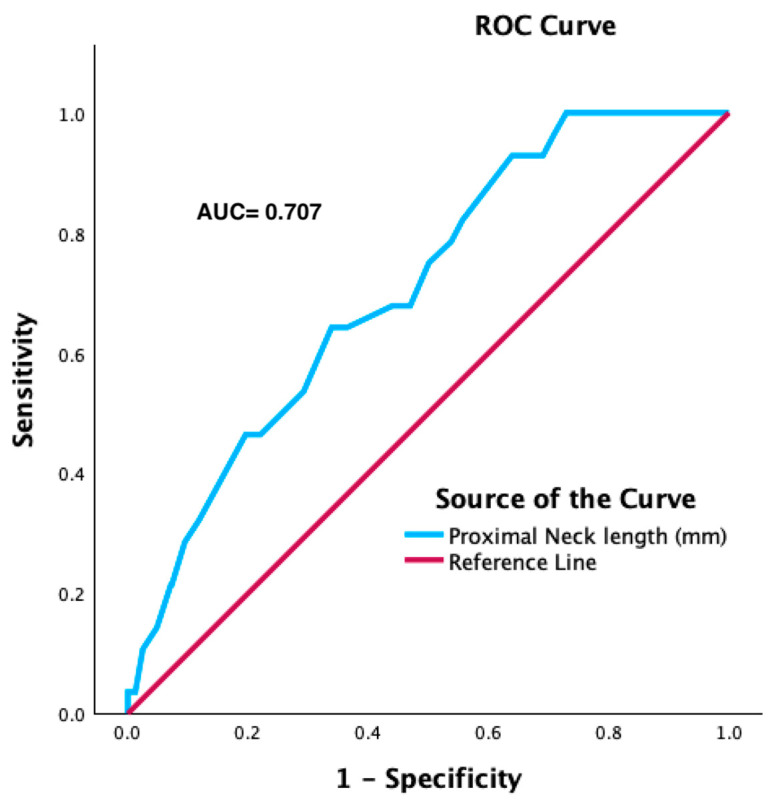
The ROC curve analysis with proximal neck length as a predictive factor for type 1A Endoleak.

ROC curve analysis identified a proximal aortic neck length of 13.5 mm as the best cut-off value for predicting the risk of type 1A endoleak during follow-up (AUC: 0.707; sensitivity: 64.3%; specificity: 33.9%) (Figure 6). At unadjusted Cox proportional hazard regression analysis, proximal aortic neck longer than 13.5 mm was associated with a significantly lower risk of type 1A endoleak (HR: 0.890; 95% CI: 0.836–0.948; *p* < 0.001).

Sac regression at least 12 months after the procedure was observed in most patients, while regression of less than 5 mm, stability, and growth combined were observed in 33.6% of patients with available data.

A sac regression of at least 10 mm at the last available follow-up is more frequently found in patients not presenting with COPD (*p* = 0.041, OR = 1.209 CI: 1.005–1.454). Sac expansion at the last available follow-up is more frequently found in patients without dyslipidemia (*p* = 0.020, OR = 1.436 CI: 1.003; 2.055)

All persistent high-flow endoleaks were treated, while for type 2 endoleaks with sac stability or reduction, a watchful waiting approach was employed. Reintervention for type 1A endoleaks consisted of proximal aortic extension with an aortic cuff (12), proximal extension with an endoanchor implant (11), suprarenal device implantation (6), and open surgery treatment in 6. Exclusion of type 1B endoleak was always performed successfully with an endovascular procedure of distal extension. All-cause mortality was reported in 36% of patients, with a non-significative higher percentage in Group 2. Survival after the procedure was 53 (IQR 25–95) months in Group 1 and 47 (IQR 25–85) months in Group 2 (*p* = 0.419).

## 4. Discussion

To the best of our knowledge, we are reporting one of the largest long-term single-center experiences with this endograft. The Endurant stent graft has not changed its core configuration since the introduction of the Endurant II endograft, and it is one of the oldest currently available stent grafts that has not transformed substantially in design over the last decade. The introduction of the Endurant IIs in 2014 enabled even more patient-tailored procedural planning with 36 limb options on both sides. This level of persistence on the market and in operating theaters around the world allowed the creation of clinical datasets based on a very large number of patients followed up for a very long time. The ENGAGE registry, initiated in March 2009, enrolled 1262 patients across 79 sites located in 30 countries [7] and is still in progress with the ENGAGE extended court, including 390 patients followed up for 10 years [8]. Since the first day of the availability of this endograft on the international market, many have hypothesized that the IFU may be too conservative and have implanted it outside of the manufacturer’s instructions in about 17% of cases between 2009 and 2011 [7]. This number is consistent with our experience. As modern adjuncts became commercially available later in our experience, our out-of-IFU cases with short, severely dilated, angulated, or tapered necks were performed without endoanchors. It is well known that out-of-IFU cases suffer from a higher overall rate of early and late complications [9]; this has also been our case with Group 2 showing a statistically significant difference for type 1A (*p* = 0.03) endoleaks and reintervention (*p* = 0.008) and a double rate, albeit without the statistical significance of type 1B endoleaks. Group 2 also showed more in-hospital days and more perioperative complications (*p* = 0.01 and 0.041, respectively), although the complications were all access-related rather than proximal-neck-related. The influence of the adherence to the IFU on sac shrinkage has been investigated for the Endurant stent graft as well as other commercially available endografts, and as in our experience, no statistically significant difference has been reported on sac shrinkage or expansion rates [10,11]. Interestingly, although the small number of aortic-related deaths makes it difficult to obtain a statistically significative difference, it appears that aneurysm-related mortality is not affected by the adherence to the IFU; it is worth considering, however, that the aneurysm-related mortality rate and the 30-days mortality rate of Group 2 is more than double that of Group 1. All-cause mortality seems not to be affected by adherence to the IFUs. Because 8 of the 9 patients who experienced post-op aortic rupture were previously lost at follow-up, follow-up enforcement seems to be the cornerstone of aortic mortality prevention, and as we went through the patients operated at our institution with whom we had lost contact, we were able to find and treat many potentially life-threatening endoleaks. The median post-op time of insurgence of complications was after 22 months (IQR 3.5; 72), and the median time between procedure and reintervention was 36 months (IQR 11; 36). These data further highlight the importance of a consistent follow-up protocol with at least yearly appointments for early detection of endograft failure. Overall freedom from any endoleak at 60 months was 89%, with 58 events, 27 of which occurred after 12 months. This is not only due to the out-of-IFU cases, as between the endoleaks reported at more than one year and less than 5, 17 occurred in Group 1, while 10 in Group 2.

Our findings may be summarized as follows:

- Out-of-IFU EVAR carries a higher risk of procedure-related reintervention and endoleak and requires a more rigorous follow-up protocol;
- Out-of-IFU EVAR does not carry higher aortic-related mortality;
- Group 2 reintervention rate is fairly low, suggesting that out-of-IFU EVAR may be used with extreme caution in selected cases;
- The Endurant stent graft proves to be reliable and is currently standing the test of time in the majority of our patients;
- A larger aneurysm carries a higher risk of endoleak;
- Some diseases or medications may have a role in influencing sac-related outcomes.

The effect of aneurysm size on endoleak risk has been investigated before on a smaller sample, and although increasing AAA size was associated with shorter overall survival, no relation was found regarding endoleak risk [12]; yet in our study, a statistically significant relation between aneurysm size and the risk of endoleak has been noted. We are currently not able to give any clinical significance to this finding as our finding needs to be confirmed on even larger datasets. Biological patient-related factors (e.g., serum metalloproteinases levels, inflammatory markers) should be considered in future studies to clarify if a bigger aneurysm means a “different” and more at-risk patient than the one presenting with a small AAA.

In our study, a proximal aortic neck longer than 13.5 mm was shown to be protective, although against type 1A endoleak. Studies on previous-generation stent grafts demonstrated that a neck shorter than 15 mm could be as safely treated as a longer one [13,14]. Presently, we tend to consider it safe to treat a patient with a proximal aortic neck between 15 and 10 mm, but a recent study found that necks shorter than 15 mm increased type 1A endoleak risk by 10.4 times [15]. Accurate assessment of the neck length required to obtain a reliable sealing, however, cannot be done without being device-specific and without a method that corrects for any errors, even millimetric, in graft placement. More studies that correct for interoperator differences and any graft positioning bias are needed to clarify this aspect.

A study from 2012 [16] states that COPD patients have more favorable endovascular outcomes, with faster sac shrinking and a lower endoleak rate, while a more recent study [17] found no statistically significant difference in COPD patients regarding sac shrinking. In our case history, patients without COPD had a higher rate of sac shrinking of more than 10 mm. This may be due to increased levels of overall inflammation found in patients presenting with COPD, as patients with altered preoperative inflammatory markers may have an altered postoperative sac behavior [18].

Our patients with dyslipidemia were found to have better endovascular outcomes. While it is well established that dyslipidemia is a risk factor for AAA development, the postoperative course of patients affected and non-affected by dyslipidemia has not been clarified yet. The effect found in our case series may be due to the use of statins or other lipid-lowering drugs that have been prescribed to all our dyslipidemic patients if they were not already taking any before surgery. Moreover, statin’s effect on the postoperative course has been described before [19,20].

Our study limitation includes the retrospective nature of the data and the absence of some follow-up data for some patients operated on a long time before data collection. More data are needed on very long-term follow-up as our current patients are proportionately younger and with fewer comorbidities and, therefore, with a longer life expectancy than the patients treated when this stent graft was first marketed [8].

## 5. Conclusions

In our experience, the Endurant stent graft is currently standing the test of time, proving safe and reliable for varying anatomies. Patients presenting with out-of-IFU proximal aortic necks have a higher rate of overall complications, reinterventions, and endoleak. Out-of-IFU procedures should be avoided or performed in selected cases, and follow-up should be enforced further for these patients. Aneurysm-related mortality, although double in the out-of-IFU treatment, shows no statistical significance. Even patients inside of the IFU but with very large aneurysms or with necks shorter than 13.5 mm tend to have worse medium and long-term outcomes and should be more carefully and more frequently evaluated to ensure early endoleak detection. Patient-related factors, such as the presence of some seemingly unrelated conditions, may affect long-term sac behavior and must be taken into account. In our experience, the scattered order of complications onset discourages remote deferral of follow-up visits, at least for certain groups of patients. A more personalized approach is required for both peri and postoperative management to ensure better outcomes, and more large datasets are needed to bring out groups with higher and lower endoleak and reintervention risks to improve patient safety and satisfaction.

## Figures and Tables

**Table 1:** Baseline demographics and comorbidities.

	Overall (795)	Group 1 (in IFU, 650)	Group 2 (Out of the IFU, 145)	*p*-Value (<0.05)
Age (years; SD)	74 ± 8	74 ± 8	75 ± 8	0.190
Female sex	63 (7.9%)	50 (7.7%)	13 (9%)	0.442
Diabetes	138 (17.4%)	114 (17.6%)	24 (16.5%)	0.798
Arterial hypertension	636 (80%)	524 (80.7%)	112 (77.2%)	0.372
Dyslipidemia	426 (53.6%)	354 (54.5%)	72 (49.6%)	0.319
Cardiac disease	335 (42.2%)	275 (42.4%)	60 (41.7%)	0.921
Previous myocardial infarction	244 (30.6%)	203 (31.1%)	41 (28.3%)	0.542
Chronic obstructive pulmonary disease	477 (59.8%)	383 (59.1%)	94 (63%)	0.650
Chronic kidney disease	192 (24.1%)	157 (24.1%)	35 (24.1%)	0.937
Ruptured aneurysm	40 (5.1%)	30 (5.1%)	10 (6.6%)	0.502

**Table 2:** Baseline anatomic features of our patients. * Statistically significant (*p* < 0.05).

	Overall	Group 1 (in IFU)	Group 2 (Out of the IFU)	*p*-Value (<0.05)
AAA diameter (mm)	61 (52; 68)	57 (50; 67)	62 (55; 74)	<0.001 *
Aortic bifurcation diameter (mm)	29 (23; 28)	29 (23; 36)	30 (25; 43)	0.025 *
Access common iliac artery diameter (mm)	15 (12; 20)	15 (12; 21)	15 (12; 19)	0.977
Access external iliac artery diameter (mm)	9 (8; 11)	9 (8; 11)	9 (8; 11)	0.119
Access femoral artery diameter (mm)	10 (9; 12)	10 (9; 12)	10 (9; 12)	0.572
Lowest renal to aortic bifurcation length (mm)	112 (101; 125)	111 (101; 124)	115 (103; 129)	0.056
Number of patent lumbar arteries	3 (2; 4)	3 (2; 4)	3 (2; 4)	0.09

**Table 3:** Baseline proximal aortic neck features of our patients. * Statistically significant (*p* < 0.05).

	Overall (795)	Group 1 (in IFU, 650)	Group 2 (out of the IFU, 145)	*p*-Value (<0.05)
Neck length	17 (12; 25)	19 (13; 26)	10 (7; 17)	<0.001 *
Neck diameter	25.1 ± 4.3	24 (22; 26)	28 (23; 33)	<0.001 *
Infrarenal neck angulation	25 (14; 42)	22 (13; 38)	35 (20; 62)	<0.001 *
Neck calcium:				
Absent	330 (41.6%)	280 (43.1%)	50 (34.4%)	0.157
<90°	380 (47.9%)	308 (47.3%)	72 (49.6%)	0.640
<180°	62 (7.9%)	48 (7.4%)	14 (9.6%)	0.404
>180°	20 (2.6%)	14 (2.1%)	6 (2.6%)	0.138
Neck thrombus:				
Absent	418 (52.6%)	343 (51.5%)	75 (52.6%)	0.756
<90°	99 (12.5%)	75 (11.5%)	24 (16.3%)	0.146
<180°	156 (19.6%)	121 (18.5%)	35 (23.5%)	0.194
>180°	121 (15.2%)	109 (17%)	12 (8.8%)	0.019 *
Neck morphology				
Straight	446 (56.1%)	409 (62.9%)	47 (32.6%)	<0.001 *
Barrel (focal <3 mm enlargement)	51 (6.3%)	48 (7.5%)	3 (2.2%)	0.042 *
Angled (>45°)	179 (22.5%)	106 (16.3%)	33 (22.5%)	<0.001 *
Bulge	9 (1.3%)	7 (1.3%)	2 (1.3%)	1.000
Conic (>10%)	134 (17.1%)	100 (15.1%)	34 (23.7%)	0.020 *
Short (<10 mm)	95 (12.1%)	27 (4.2%)	68 (47.1%)	<0.001 *
Dilated (>28 mm)	228 (29%)	149 (22.9%)	79 (54.3%)	<0.001 *
Irregular (elliptic shape)	23 (3%)	17 (2.6%)	6 (4.4%)	0.256

**Table 4:** Periprocedural data. * Statistically significant (*p* < 0.05).

	Overall	Group 1 (in IFU)	Group 2 (out of the IFU)	*p*-Value (<0.05)
Length of stay (days)	4 (3; 6)	4 (3; 6)	5 (4; 6)	0.01 *
Procedure duration (hours)	3.2 (2.3; 4.3)	3.2 (2.3; 4.2)	3.3 (2.45; 4.5)	0.221
Sac embolization	184 (23.2%)	149 (22.9%)	35 (24.1%)	0.827
Access or procedural Complications	50 (6.3%)	35 (5.5%)	15 (10.3%)	0.041 *
Number of Endurant components	3 (2; 3)	2 (2; 3)	3 (2; 3)	0.486
Bell-bottom	224 (28.2%)	181 (27.9%)	43 (29.8%)	0.824
Proximal Oversize	1.14 (1.06; 1.23)	1.14 (1.06; 1.23)	1.12 (1.05; 1.23)	0.381

**Table 5:** Outcomes and sac regression, including all patients. * Statistically significant (*p* < 0.05).

	Overall (795)	Group 1 (in IFU) (650)	Group 2 (out of the IFU) (145)	*p*-Value (<0.05)
30-days death	29 (3.7%)	20 (3.1%)	9 (6.2%)	0.070
Follow-up time (months)	43 ± 39	43 ± 40	40 ± 37	0.582
Any endoleak	82 (10.5%)	60 (9.4%)	22 (15.3%)	0.037 *
Type 1A	34 (4.3%)	23 (3.5%)	11 (7.6%)	0.030 *
Type 1B	19 (2.4%)	13 (2%)	6 (4.1%)	0.129
Type 2	30 (3.8%)	25 (3.9%)	5 (3.4%)	0.523
Graft thrombosis	17 (2.1%)	14 (2.2%)	3 (2.1%)	0.947
Procedure-related reintervention	83 (10.4%)	59 (9.1%)	24 (16.6%)	0.008 *
Aneurysm-related mortality	9 (1.1%)	6 (0.9%)	3 (2.1%)	0.377
All-cause mortality	290 (36.5%)	229 (35.2%)	61 (42.1%)	0.328
Sac regression at more than one year:	N = 509	N = 404	N = 105	
≥10 mm	46.3%	44.6%	54.1%	0.178
≥5 mm	65.9%	64%	73.8%	0.148
<5 mm/stable	21.3%	22.8%	14.8%	0.164
Growth	13.3%	13.4%	12.9%	0.912

## Data Availability

Further inquiries may be directed to the corresponding author.
